# A Social Media Analysis of Pemphigus

**DOI:** 10.2196/50011

**Published:** 2023-10-19

**Authors:** Gaurav Nitin Pathak, Rithi John Chandy, Vidisha Naini, Shazli Razi, Steven R Feldman

**Affiliations:** 1 Department of Dermatology Rutgers Robert Wood Johnson Medical School Somerset, NJ United States; 2 Department of Dermatology Wake Forest University School of Medicine Winston-Salem, NC United States; 3 Department of Dermatology Rao Dermatology Atlantic Highlands, NJ United States; 4 Department of Internal Medicine Jersey Shore University Medical Center Neptune, NJ United States; 5 Department of Pathology Wake Forest University School of Medicine Winston-Salem, NC United States; 6 Department of Social Sciences and Health Policy Wake Forest University School of Medicine Winston-Salem, NC United States; 7 Department of Dermatology University of Southern Denmark Odense Denmark

**Keywords:** pemphigus, social media, pemphigus vulgaris, Facebook, YouTube, Twitter, Instagram, dissemination, medical information, autoimmune disease, diagnosis, engagement, educational, content, awareness

## Abstract

An analysis of the pemphigus content on Facebook, Twitter, Instagram, and YouTube social media platforms.

## Introduction

In 2021, an estimated 4.26 billion people reported using some type of social media including approximately 80% of dermatology patients [1]. Social media has advanced health research and practice, enhanced social mobilization, and facilitated health services and events [2]. Approximately 61% of US adults utilize the web for health-related information, most commonly for diseases and treatments [3].

Pemphigus represents a spectrum of autoimmune skin-blistering diseases, with a prevalence of 5.2 cases per 100,000 adults, and is associated with diagnostic delay [4,5]. Social media may be used to shorten diagnostic delays, disseminate disease information, and connect affected individuals to support groups. The purpose of this study is to characterize the most popular and recent social media footprint of pemphigus across common social media platforms.

## Methods

Four social media platforms—Facebook, Instagram, YouTube, and Twitter—were evaluated using the search term “pemphigus.” Data collection was conducted at singular date cutoff timepoints to collectively evaluate the most recent and popular social media content. Only English content related to human pemphigus was included. The exclusion criteria were posts that discussed nonhuman pemphigus, non-English content, and YouTube videos longer than 20 minutes. The Quality Evaluation Scoring Tool (QUEST) score is a validated metric used to analyze the quality of medical content posted on the web and was used to evaluate content on YouTube. Details regarding the data collection process are available in Multimedia Appendix 1.

## Results

### YouTube

Of the 10 identified eligible YouTube videos, 5 (50%) were made by physicians, 4 (40%) by various organizations, and 1 (10%) by patients (Table 1). All videos were educational, and the average length of the videos was 4 minutes and 42 seconds. The average number of views per video was 23,404, and the average number of likes and comments was 411 (SD 653) for each video. The average QUEST score for the selected videos was 14.6 (SD 4.1; Table 1).

**Table 1:** Analysis of top YouTube, Instagram, and Twitter content.

Author or content category		Views/posts^a^, n	Post sender or type of content, n (%)		Engagement^b^		QUEST^c^ score, mean (SD)		
**Top 10 YouTube videos**				N/A^d^					
	1. Physician, educational	50,718			601		19 (1.4)		
	2. Patient/organization, personal	1726			Not disclosed		9.5 (2.1)		
	3. Health care professional, educational	7222			150		11 (0.0)		
	4. Physician, educational	4367			37		19.5 (3.5)		
	5. Physician, educational	171			2		20 (2.8)		
	6. Organization, educational	115,321			1983		13.5 (0.7)		
	7. Organization, educational	3442			36		9.5(0.7)		
	8. Organization, educational	44,549			781		13 (0.0)		
	9. Physician, educational	2844			60		18 (0.0)		
	10. Organization, educational	3677			50		12.5 (0.7)		
**Top 50 Instagram post**									N/A
	Promotional	17	Organization: 13 (76) Physician/Professor: 4 (24)		Likes, n: 240 Likes, mean (SD): 14.1 (11.6) Comments, n: 13				
	Educational	15	Organization: 9 (60) Physician: 3 (20) Patient: 3 (20)		Likes, n: 321 Likes, mean (SD): 21.4 (32.3) Comments, n: 8				
	Recruitment	16	Organization: 16 (100)		Likes, n: 151 Likes, mean (SD): 9.44 (5.2) Comments, n: 5				
	Personal	2	Patient: 2 (100)		Likes, n: 33 Likes, mean (SD): 16.5 (19.5) Comments, n: 18				
**Top 50 Twitter posts**									N/A
	Physician	25	Educational: 16 (64) Personal: 7 (28) Promotional 2 (8)		Total: 1608 Int/Post^e^, mean (SD): 64.3 (178.7)				
	Patients/individuals	3	Personal: 2 (67) Educational: 1 (33)		Total: 10 Int/Post, mean (SD): 3.3 (0.6)				
	Organization	15	Educational: 9 (60) Personal: 2 (13) Promotional: 3 (20) Recruitment: 1 (7)		Total: 101 Int/Post, mean (SD): 6.7 (5.8)				
	Pharmaceutical company	1	Recruitment: 1 (100)		Total: 25 Int/Post: 25				
	Promoter	2	Promotional: 2 (100)		Total: 0 (17 video views) Int/Post: 0				
	Researcher	4	Educational: 2 (50) Personal: 1 (25) Promotional: 1 (25)		Total: 54 Int/Post, mean (SD): 13.5 (17.3)				

^a^For YouTube, this column is a count of views for each video. For Instagram and Twitter, this column is the count of posts for each category.

^b^For YouTube, engagement is the total number of likes + comments for each video. For Instagram, engagement includes the total number of likes and comments and the average number of likes for each category of post. For Twitter, engagement includes the total number of likes + retweets + comments, as well as the average interactions per post.

^c^QUEST: Quality Evaluation Scoring Tool.

^d^N/A: not applicable.

^e^Int/Post: interactions per post.

### Instagram

A total of 49 Instagram posts were excluded. Of the 50 included eligible posts, 17 (34%) were categorized as “promotional,” 15 (30%) were “educational,” 16 (32%) were “recruitment,” and 2 (4%) were “personal” (Table 1). Organizations were the most common post senders (n=38, 76%) and contributed the majority of promotional (13/17, 77%), educational (9/15, 60%), and recruitment (16/16, 100%) posts (Table 1).

### Twitter

Of the 50 tweets identified, approximately 39 (78%) included images, 3 (6%) had videos, and 8 (16%) were only text. Physicians were the most common tweet senders, with 25 (50%) tweets and the highest average engagement (64.3, SD 178.7 interactions/post). The majority of posts were educational (n=29, 58%).

### Facebook

The majority of the top Facebook groups were private and focused on pemphigus vulgaris support (8/10 groups) with the top 3 Facebook groups having over 1000 members each (Table 2). Of the 25 identified posts, individual posts were the most common (n=17, 68%), while posts made by patients/caregivers generated the highest average engagement (272.2, SD 264.7 interactions/post).

**Table 2:** Analysis of Facebook support group and post content.

			Access type		Posts, n			Members, n			Type of content, n (%)			Activity			Total engagement (likes/reactions + shares + comments) and Int/Post^a^	
**Top 10 Facebook support groups**						N/A^b^						N/A						N/A
	1. Pemphigus Vulgaris	Private^c^					4900						4 posts/day					
	2. Pemphigus Vulgaris	Private					2300						9 posts/week					
	3. Pemphigus Vulgaris Support and Awareness	Private					1400						2 posts/month					
	4. Pemphigus Vulgaris	Public					112						0 posts					
	5. Pemphigoid and Pemphigus Nation	Public					244						8 posts/year					
	6. Living with Pemphigus Foliaceus	Private					350						2 posts/week					
	7. Pemphigus Vulgaris in India	Private					300						1 post/month					
	8. Pray4Elyse MCD Castleman’s Disease/Paraneoplastic pemphigus	Public					90						3 post/year					
	9. Pemphigus and Pemphigoid Australia/NZ	Private					90						0 posts/week					
	10. Pemphigus Vulgaris Victoria	Private					4						2 posts/month					
**Top 25 Facebook posts**			N/A					N/A						N/A				
	Physician			0						N/A						Total: 0 Int/Post: 0		
	Patient/caregiver			5						Personal: 4 (80) Educational: 1 (20)						Total: 1361 Int/Post, mean (SD): 272.2 (264.7)		
	Individual			17						Awareness: 16 (94) Educational: 1 (6)						Total: 432 Int/Post, mean (SD): 25.4 (17.7)		
	Organization			2						Educational 2 (100)						Total: 359 Int/Post, mean (SD): 179.5 (248.2)		

^a^Int/Post: interactions per post.

^b^N/A: not applicable.

^c^Private groups require admin approval before content can be accessed by the user.

## Discussion

### Principal Findings

Social media provides an avenue for physicians, patients, and organizations to share educational, personal, and promotional content to improve rare disease awareness [6]. Approximately half of the YouTube videos were made by physicians, yet content made by organizations had the highest engagement. The average QUEST score (14.6, SD 4.1) across the analyzed YouTube videos was higher than those for other dermatologic conditions, suggesting higher quality content [7].

Instagram had the highest portion of nonhuman–related pemphigus content, highlighting a need for more reliable human-related pemphigus information. Although Twitter has the highest rate of medical misinformation, half of the top filtered posts were made by physicians, and the majority of posts were educational [8].

Social media has enhanced clinical trial recruitment, and given the rarity of pemphigus, social media can improve awareness of ongoing clinical trials [9]. However, Twitter and Instagram are the only identified platforms with recruitment posts (2/50, 4% and 16/50, 32%, respectively). Additionally, Facebook groups allow patients to connect with others to discuss disease-related concerns and resources.

### Limitations and Future Directions

The limitations of this study include that our data was collected at a singular time point for each platform with largely descriptive data. Additionally, other platforms such as TikTok, Snapchat, WhatsApp, and Reddit were not analyzed. Future studies should evaluate the accuracy of medical content and implications of misinformation posted on the web.

### Conclusion

Current uses of social media for pemphigus revolve around better understanding the disease, developing support groups, and improving awareness. Physicians can use these avenues to connect patients globally to discuss their experiences. Social media also offers a platform for greater clinical trial recruitment for those with rare diseases.

## Appendix

Multimedia Appendix 1 Supplementary methods.

## Abbreviations

QUEST: Quality Evaluation Scoring Tool
