# Supplementary material for: A Social Media Analysis of Pemphigus
Source: JMIR Dermatol. 2023 Oct 19;6:e50011. doi: 10.2196/50011 (PMC10623221; doi:10.2196/50011)
Supplement: Multimedia Appendix 1 [file derma_v6i1e50011_app1.docx]

**Supplemental methods:**

Due to the day-to-day variability of content, data from Instagram, Twitter, and Facebook was collected on April 17th, 2023 by reviewers G.N.P and V.N. Data from YouTube was collected on a different date (May 9th, 2023) since YouTube’s most popular videos tend to stay stable, and due to availability of authors. Social media applications with high variability and a lot of content such as Instagram and Twitter had more overall content evaluated to better represent the type of pemphigus content discussed. Platforms such as YouTube and Facebook had less variability and less overall relevant content so fewer content was evaluated to best represent the true pemphigus footprint on that platform.

**YouTube**

Using the search phrase “pemphigus”, the top 10 video results filtered by relevance that met inclusion criteria and were less than 20 minutes were analyzed. Data collected included video title, author background (physician, patient, organization, other), type of pemphigus, type of information presented (educational, promotional, personal, other), length of video, number of views, date of publication, number of likes, number of comments and QUEST score. To assess the QUEST score, two of the authors scored the videos individually using the QUEST tool, which is a validated metric used to assess medical content posted online. An average of the QUEST scores were posted. Searches were cross-checked by two individuals and all data collection occurred in one day.

**Instagram**

Using the search phrase #pemphigus, the most recent top 50 posts as per the Instagram algorithm that met study inclusion criteria were analyzed. Data collected included Instagram handle, sender background (physician, patient, organization, other), date of post, type of post (educational, promotional, personal, other), type of post (video, picture and/or text), number of likes, number of comments, and number of views. Searches were cross-checked by two individuals and all data collection occurred in one day. The number of posts that did not meet the inclusion criteria as well as the reason for exclusion were also recorded.

**Twitter**

Using the search phrase #pemphigus, the most relevant top 50 tweets as per the Twitter algorithm that met study inclusion criteria were analyzed. Data collected included tweet sender handle, sender background (physician, patient, organization, other), type of content presented (educational, promotional, personal, other), type of tweet (video, image, and/or text), date of post, number of likes, number of comments, number of retweets, number of tweet views (when applicable), and number of video views (when applicable). Searches were cross-checked by 2 individuals and all data collection occurred in one day. The number of tweets that did not meet the inclusion criteria as well as reason for exclusion were also recorded.

**Facebook**

Facebook searches of both groups and posts were conducted. The top 10 support groups and most recent 25 posts in 2022 were used for data collection.

*Groups:*

Using the search phrase “pemphigus”, the most relevant top 10 groups as per Facebook algorithm that met study inclusion criteria were analyzed. Data collected included name of group, type of access (public or private), date of group creation, number of current members, demographics of members (if applicable), activity (number of posts per day/week), and level of activity (posts/time). Searches were cross-checked by 2 individuals and all data collection occurred in one day. To protect privacy, we did not request membership for those Facebook groups that were private, although we did still collect any publicly reported information on the group home page.

*Posts:*

Using the search phrase “pemphigus”, the most recent 25 posts as per the Facebook algorithm in 2022 that met study inclusion criteria were analyzed. Data collected included name of post sender, sender background (physician, patient, organization, other), type of content (educational, promotional, personal, other), date of post, type of post (video, image and/or text), number of likes/reactions, number of comments, and number of shares. The number of posts that did not meet inclusion criteria were also obtained and recorded.
